# Effect of Promoter Polymorphisms on Cytokine Concentration in Preterm Breast Milk and Subsequent Infant Outcomes

**DOI:** 10.1177/0890334416646725

**Published:** 2016-06-01

**Authors:** Kelley L. Baumgartel, Maureen W. Groer, Susan M. Cohen, Dianxu Ren, Diane L. Spatz, Yvette P. Conley

**Affiliations:** 1 University of Pittsburgh, School of Nursing, Pittsburgh, PA, USA; 2 University of South Florida, College of Nursing, Tampa, FL, USA; 3 University of Pennsylvania School of Nursing, Philadelphia, PA, USA

**Keywords:** breastfeeding, breast milk, calprotectin, interleukin, preterm infants, single nucleotide polymorphism, trajectory analysis

## Abstract

**Background:**

Breast milk concentrations of immune components are variable between women and interleukin (IL) differences may be associated with infant outcomes. Molecular mechanisms for milk variability remain unknown.

**Objective:**

The aims were to (1) examine the relationship between maternal IL genotypes and milk concentrations of IL4, IL6, and IL10, (2) describe the trajectories of milk IL change, (3) examine whether maternal IL genotypes predict IL trajectories and/or average weekly IL concentration, and (4) examine if weekly IL levels and/or IL trajectories are associated with infant outcomes.

**Methods:**

Milk aliquots were collected from each feeding of mother's own milk and pooled weekly. DNA was extracted from 1 sample of each mother's breast milk whey (n = 64), and single nucleotide polymorphisms (SNPs) of IL genes were genotyped. Milk IL concentrations were measured and trajectory analysis examined IL milk change over time. Multivariate breast milk IL concentration analyses controlled for gestational age and prepregnancy body mass index. Multivariate infant outcome (n = 73) analyses controlled for gestational age and the ratio of human milk to total milk.

**Results:**

Trajectory analysis resulted in linear group shapes, with 2 distinct subgroups in IL6 and 3 subgroups in IL4 and IL10. Trajectory groups trended toward significance with calprotectin, intraventricular hemorrhage, and blood transfusions. Multivariate analyses resulted in trending associations between maternal SNPs and subsequent IL6 and IL10 milk levels. There was a trending relationship between IL milk levels and both fecal calprotectin and intraventricular hemorrhage.

**Conclusion:**

Maternal IL SNPs may affect IL breast milk levels and IL milk levels may be associated with infant outcomes.

## Well Established

Serum interleukin (IL) levels are influenced by single nucleotide polymorphisms (SNPs). Breast milk IL concentrations are highly variable between women and may be associated with disparate infant outcomes.

## Newly Expressed

This study is the first to show a relationship between maternal IL SNPs and subsequent IL breast milk concentration. Variable milk IL levels were also associated with preterm infant outcomes.

## Background

Preterm birth complications are the leading cause of death among children younger than 5 years.^1^ Preterm infants face increased risks of pneumonia, retinopathy, necrotizing enterocolitis (NEC), and sepsis. Breast milk provides protection against these complications,^2-7^ and for this reason, the American Academy of Pediatrics recommends that all infants, particularly those weighing less than 1500 grams at birth, receive human milk.^8^ The robust immunological profile of breast milk may contribute to this protective influence on vulnerable infants.

Preterm infants no longer receive maternal immunological protection in utero; therefore, it is essential that they receive passive immunity through breast milk. Interleukins (ILs) are an integral part of the inflammatory response, and preterm infants are vulnerable to complications due, in part, to an underdeveloped immunological system. Interleukins are present in breast milk, although their concentration is highly variable between women.^9^ Breast milk from mothers with allergies is higher in IL4 concentration when compared to milk from mothers without allergies.^10,11^ IL4 is relevant to the preterm population because serum IL4 induces both antibody and IgE production,^12^ although it is unclear if exogenous milk IL4 has this effect on the breastfed infant. There is some evidence that varying IL4 in breast milk may contribute to the development of allergic dermatitis in healthy term infants.^13^ Serum IL4 levels have been associated with 2 single nucleotide polymorphisms (SNPs) at the gene's promoter region,^14,15^ although this relationship has never been explored in breast milk.

IL6, a pleiotropic cytokine, promotes the growth of monoclonal antibodies. IL6 is poorly regulated in preterm infants^16^; furthermore, higher milk IL6 levels are associated with decreased infant weight gain, percentage fat, and fat mass among healthy breastfed infants,^17^ although this work was exploratory and included a small sample size (n = 19). Two SNPs, also in the regulatory region, have been implicated in varying IL6 levels,^18^ although its effect on milk concentration has never been explored.

The role of IL10 in the prevention of inflammatory pathologies, as well as its function as a feedback regulator,^19^ illustrates its importance to preterm infants who are vulnerable to infection. IL10 concentration in breast milk is higher at 3 months postpartum among mothers who report allergies.^20^ Variable levels of IL10 in breast milk have been implicated in disparate neonatal outcomes, including immeasurable IL10 in milk fed to infants who developed NEC^21^ and higher milk levels associated with neonatal jaundice.^22^ IL10 is influenced by SNPs in the gene's promoter region^23-26^; however, the effect of SNPs on milk IL10 levels has never been examined.

Breast milk composition is influenced by gestational age at delivery and lactation stage, with higher cytokine milk levels that increase during lactation among preterm milk compared with term milk.^27^ This pattern of increasing cytokine concentration over time may explain why mother's own milk (MOM) is highly protective against infection, particularly NEC. Trajectory analysis of human milk ILs may illustrate the dynamic nature of breast milk, as this approach allows for estimation of multiple groups within a population, as opposed to a traditional regression that models only 1 mean within the population.^28^

The association between maternal SNPs and breast milk IL concentration has never been explored, despite evidence that milk IL concentration is variable between women. IL4, IL6, and IL10 are present in milk at highly variable amounts between women. Although limited, exploratory research suggests that milk IL variability may be associated with several infant outcomes. The purpose of this study was to, over the first 3 weeks postpartum and in a population who delivered preterm, (1) examine the relationship between maternal IL genotypes and cross-sectional (weekly) breast milk concentrations of IL4 (rs2243250 -589 T/C, rs2070874 -33 T/C), IL6 (rs1800795 -174 C/G, rs1800796 -572 G/C), and IL10 (rs1800871 -819 C/T, rs1800872 -592 A/C, rs1800896 -1082 G/A), (2) describe the trajectories of breast milk IL concentration change over time, (3) examine whether maternal IL genotypes are associated with breast milk IL trajectories, and (4) examine if weekly IL levels and/or IL trajectories predict infant outcomes.

## Methods

### Study Population

This ancillary study included women (n = 64) who delivered infants (n = 73, including multiples) with a birth weight < 1500 grams who were admitted to a level 3 neonatal intensive care unit (NICU) between 2011 and 2014. We were able to collect genotype data from DNA in prospectively collected breast milk samples over the first 3 weeks postpartum. Mothers with HIV, infants with major congenital anomalies, and moribund infants were excluded. Informed consent was obtained from all subjects and this study was approved by the University of Pittsburgh's Institutional Review Board.

The following variables were collected and available for analysis: maternal age, income, education, ethnicity, race, marital status, and pregnancy history. Medical record data provided information about the labor and delivery of the infant(s). Maternal prepregnancy weight was self-reported, and the height was obtained from the medical chart. The following equation, recommended by the Centers for Disease Control and Prevention, was used to obtain a body mass index (BMI) for each participant: weight (pounds) / [height (inches)]^2^ × 703. Infant data were obtained from the NICU medical record and included gender, ethnicity, gestational age at birth, birth weight, APGAR scores, ratio of MOM to total milk, and SNAP scores (illness severity). Continuous outcomes included length of stay, weight gain at 6 weeks, days on oxygen, and fecal calprotectin (see below). Categorical outcomes (yes/no) included sepsis, retinopathy of prematurity (ROP), NEC, intraventricular hemorrhage (IVH), blood transfusions, and feeding intolerance.

### Breast Milk Collection and Whey Separation

Any volume and source of milk that the infant received (including MOM, donor breast milk, and formula) was recorded from the infant's medical record. Breast milk aliquots from each feeding were collected for up to 3 weeks and these aliquots were pooled weekly. All milk was collected and stored frozen until brought to the laboratory twice weekly. The pooled MOM was centrifuged, defatted, and filtered, and the whey was frozen at −80°C. Although infants received both donor milk and MOM, only MOM was examined and genotyped for this study.

### Interleukin Measurement

Interleukin concentrations were measured using a bead-based assay on a MagPix instrument (Luminex) and Millipore kits (Emd Millipore) and are detailed in previous work.^29^ Interleukin concentrations were measured weekly in all pooled samples of MOM for the first 3 weeks postpartum. Each assay included a standard curve and quality controls, and all samples were done in duplicate. Mean intra-assay coefficients of variation for all cytokines ranged from 3.9% to 6.9%.

### Fecal Calprotectin

Fecal calprotectin has been used as a biomarker of inflammation within the preterm population, as calprotectin is an indicator of neutrophil migration.^30^ Calprotectin levels were measured in weekly stool samples using the PhiCal™ Fecal Calprotectin Immunoassay (Geneva Diagnostics). Quality controls were done for all fecal calprotectin assays. Intra-assay coefficient of variation was 7.7%.

### Genotyping

DNA was extracted from maternal breast milk whey using the Qiagen DNA Extraction Mini Kit. Genotype data were collected using TaqMan allele discrimination assays to genotype 7 functional promoter polymorphisms of IL4 (rs2070874, rs2243250), IL6 (rs1800795, rs1800796), and IL10 (rs1800871, rs1800872, rs1800896). We performed TaqMan allelic discrimination with the ABI Prism 7000 Sequence Detection System and SDS software v1.2.3 (Applied Biosystems Inc). Negative controls were included and a portion of the samples was repeated to confirm that they repeatedly discriminated into the same genotype. We also included duplicates and performed independent blinded double calls, and discrepancies were regenotyped. Blinded raw data were reexamined for the SNPs for which Hardy-Weinburg Equilibrium (HWE) was violated to rule out genotyping error.

### Statistical Analysis

#### Preliminary analysis

Statistical analyses were performed using SAS (version 9.4). Univariate outliers were assessed using both frequency tables and graphical methods. Multivariate outliers were assessed using scatterplots. Missing data were assessed for both amount and pattern. Normality was assessed with the Shapiro-Wilks test and graphically at each time point. Data transformations were performed when a regression assumption was compromised. There were 8 sets of multiples, including 1 set of triplets. Milk-specific analyses excluded 1 twin, or 2 triplets, removed randomly, to ensure that each mother was represented only once (n = 64). For infant-specific aims, all infants were included in the analyses (n = 73).

#### Trajectory modeling

Trajectory modeling (PROC TRAJ) with the censored normal model was used to examine changes in breast milk. When determining the number of trajectory groups, all group orders were set to second order when fitting the maximum number of groups.^31^ After the number of groups was determined, trajectory shapes were evaluated in a stepwise manner, up to a second order polynomial. Participants were assigned to a trajectory group based on their highest posterior group probability. We compared Bayesian Information Criteria (BIC) between the models to determine the appropriate number of groups and trajectory weights.

#### Univariate analyses

Univariate analyses were performed for each association, and any relationship with a *P* value ≤ .20 was included in the multivariate model. Due to a small sample size and the need for ethnic subset analyses, which further decreased sample size, SNP-specific analyses included minor allele absence, with the exception of rs2243250. The minor alleles for rs2243250 are inconsistent between the represented ethnicities in this sample; therefore, genotypes were included in the rs2243250 analysis. The Fisher exact test was used to examine the relationships between (1) SNP and categorical infant outcomes, (2) SNP and IL trajectory group, and (3) IL trajectory group and categorical infant outcomes.

#### Multivariate analyses

We considered *P* values ≤ .05 significant, although we also considered trending relationships (*P* ≤ .10) in our discussion due to (1) the exploratory nature of a pilot study, (2) small sample size, and (3) race-specific subgroup analyses, which further decreased our sample size. Multivariate breast milk IL concentration analyses controlled for both gestational age and prepregnancy BMI. Multivariate infant outcomes analyses controlled for gestational age and ratio of MOM to total milk. Infant outcomes were examined using a multivariate approach only when there was a trending or significant relationship (*P* ≤ .10) between maternal SNP and IL milk concentration in the multivariate analysis. The association between maternal SNPs and IL trajectory grouping was examined without covariates because this relationship has never been explored. Effect sizes (semipartial omega squared [ω_p_^2^]) were calculated for continuous outcomes and odds ratios were calculated for categorical outcomes. Multivariate models that included the total population were examined using both minor allele absence and genotype. Continuous outcomes were examined using multiple linear regression and binary outcomes were examined with multiple logistical regression. The relationship between trajectory group and continuous infant outcomes was assessed by generating multiple contingency tables using IL trajectory group and outcomes. We also performed general linear regression models to measure these associations, since a sample size of at least 100 is ideal to perform trajectory analyses.^31^

## Results

### Demographics and HWE

The average maternal age was 28.3 (± 6.8) years, the sample was mostly African American (39.68%) and multiparous (mean of 3 pregnancies ± 2.4), and the average prepregnancy BMI was 27.8 (± 7.3) (Tables 1 and 2). There were 3 SNPs not in HWE: rs2070874, rs2243250, and rs1800796 (data not shown). The infants were born at, on average, 28.3 (± 2.4) weeks gestation and weighed 1069.6 (± 216.8) grams. APGAR scores at 1 minute were, on average, 5.9 (± 1.9) and at 5 minutes, 7.4 (± 1.5). SNAP scores averaged 2.4 (± 0.5), they required oxygen for 15.2 (± 21.3) days, and their length of stay in the NICU was 70.5 (± 37.0) days.

### Interleukin Trajectory Modeling

**Figure 1:**
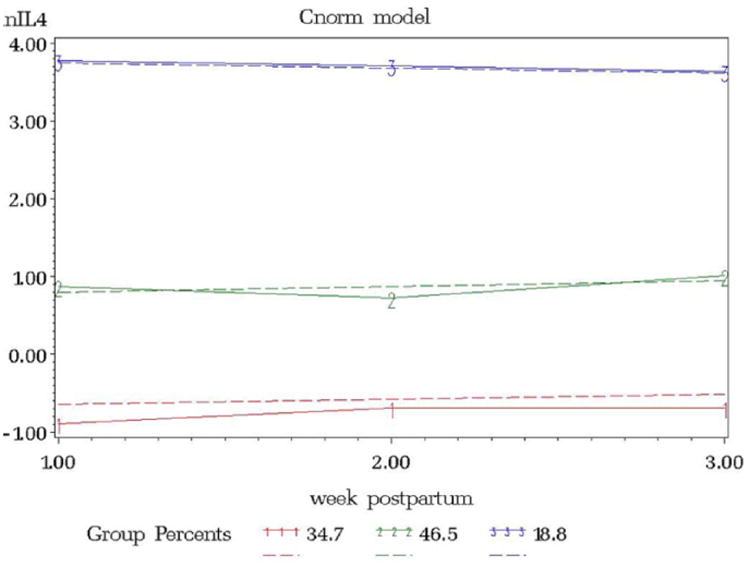
lnIL4 versus Week Postpartum.

**Figure 2:**
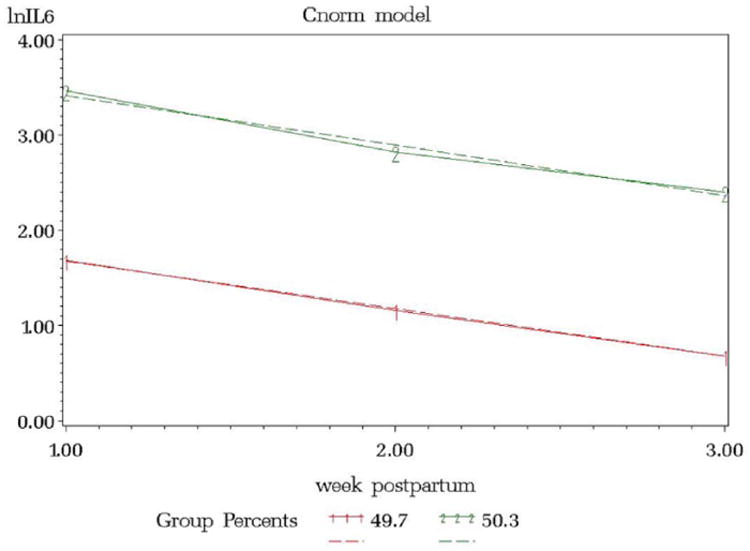
lnIL6 versus Week Postpartum.

**Figure 3:**
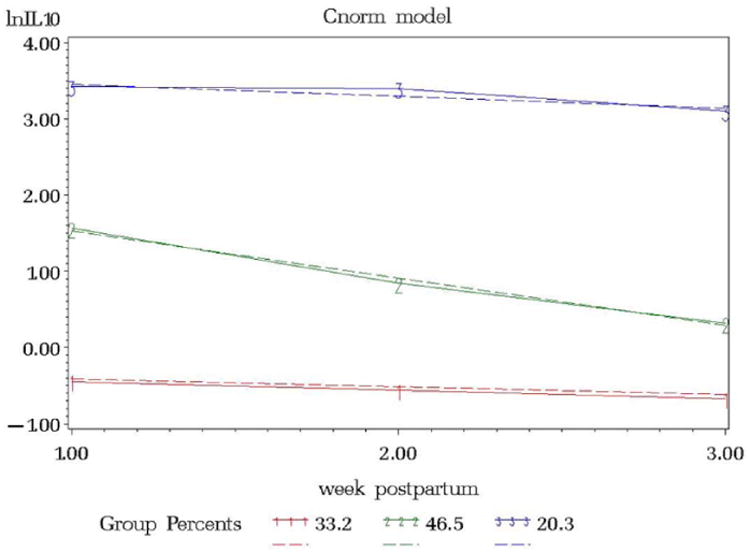
lnIL10 versus Week Postpartum.

Interleukin levels were natural log transformed to fulfill the normality assumption. The resulting model from the IL4 trajectory model included 3 groups, ordered (1) low linear (34.7%), (2) middle linear (46.5%), and (3) high linear (18.8%) (Figure 1). The IL6 trajectory resulted in 2 groups, ordered (1) low linear (49.7%) and (3) high linear (50.3%) (Figure 2). IL10 resulted in 3 groups, ordered (1) low linear (33.2%), (2) middle linear (46.5%), and (3) high linear (20.3%) (Figure 3).

### Maternal Interleukin SNPs and Breast Milk Interleukin Concentration/Interleukin Trajectory

**Figure 4:**
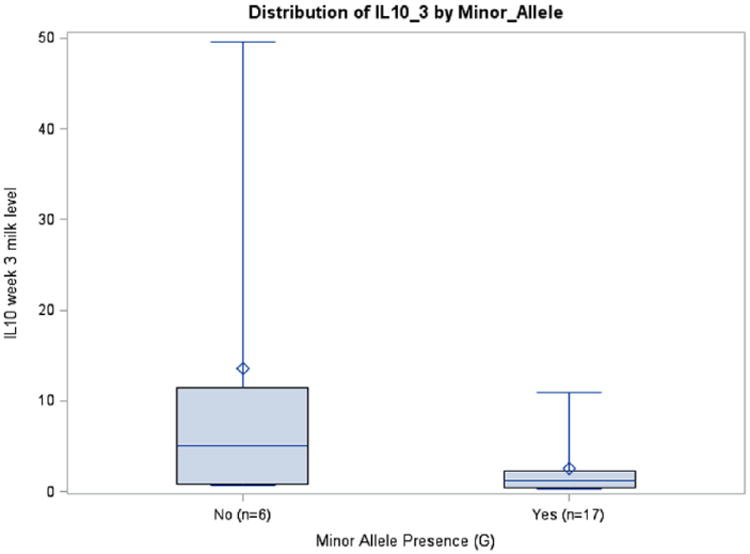
IL10 Week 3 Milk Levels with and without Minor Allele rs1800896 (*P* = .0705), African Americans.

**Figure 5:**
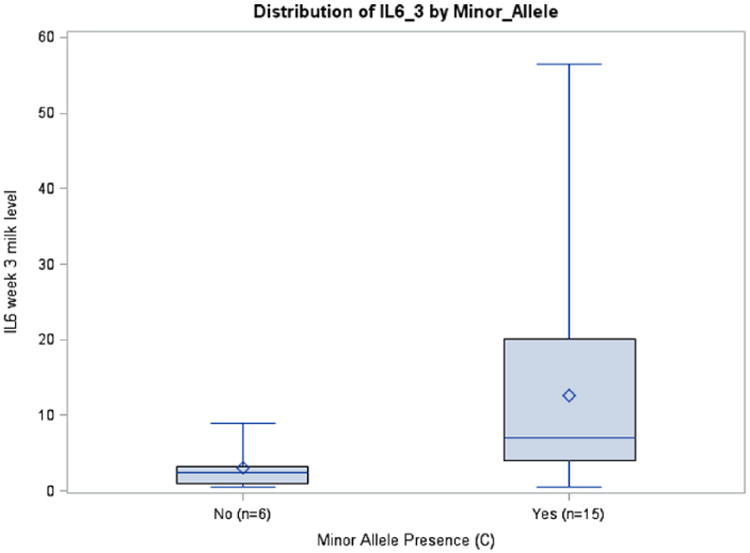
IL6 Week 3 Milk Levels with and without Minor Allele rs1800795 (*P* = .097), Caucasians.

**Figure 6:**
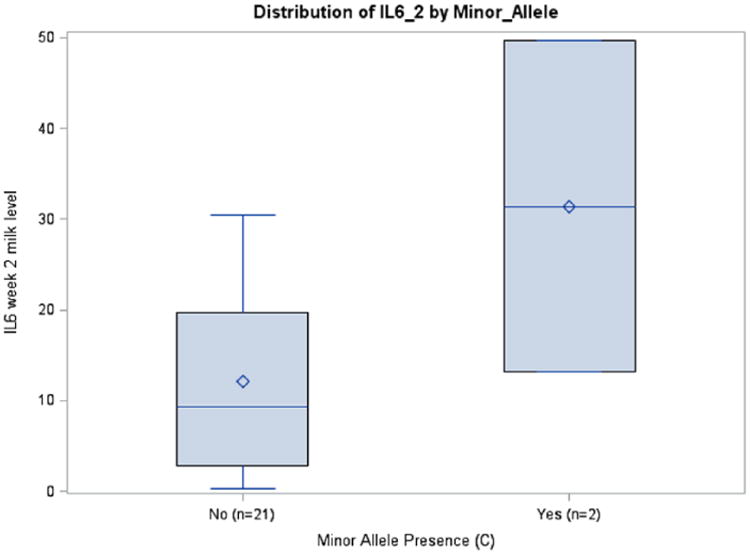
IL6 Week 2 Milk Levels with and without Minor Allele rs1800796 (*P* = .0772), African Americans.

Prepregnancy BMI was inversely associated with IL6 milk concentrations in the rs1800795 model for week 1 (*P* = .0411) and average IL6 (*P* = .0126), although this was seen only in African Americans. A similar association among African Americans was observed between BMI and IL6 milk concentration in the rs1800796 model for week 2 (*P* = .0288) (Table 3). Although there were no significant relationships between rs1800871 minor allele absence and IL10 milk concentration, BMI was inversely associated with milk IL10 levels at week 2 (*P* = .0350) and average IL10 (*P* = .0263). Body mass index was also related to IL10 milk concentration when examining minor allele absence of rs1800872 at week 1 (*P* = .0260), week 2 (*P* = .0250), and average IL10 (*P* = .015). Absence of minor allele rs1800896 was nearly significantly associated with milk IL10 levels at week 3 among African Americans (*P* = .0705, ω_p_^2^ = 0.1145) (Table 3; Figure 4). There was a trend toward significance between absence of minor allele for rs1800795 and IL6 milk concentration among Caucasians at week 3 (*P* = .0966, ω_p_^2^ = 0.1002) (Table 3; Figure 5). African Americans have a trend toward significance between rs1800796 minor allele absence and IL6 milk levels at week 2 (*P* = .0772, ω_p_^2^ = 0.0477) (Table 3; Figure 6). After controlling for gestational age and maternal BMI, there were no significant associations between IL4 genotypes and IL4 breast milk concentrations (data not shown).

### Interleukin Trajectory, Interleukin Concentration, and Infant Outcomes

After controlling for gestational age and ratio of MOM to total milk received, there was a significant association between IL6 group 1 membership and IVH (odds ratio [OR] = 6.275; 95% confidence interval [CI], 1.076-36.584; *P* = .0412) (Table 4) and a trend toward significance with fecal calprotectin at week 3 (*P* = .0822, ω_p_^2^ = 0.0376) (Table 5). Infants who received breast milk from IL4 group 2 were 4.16 times more likely to receive a blood transfusion when compared with infants who received breast milk from trajectory group 3 (OR = 4.162; 95% CI, 0.778-22.277; *P* = .0712) (Table 4). There was a trending relationship between IL6 milk levels at week 2 and calprotectin at week 3 (*P* = .0978, ω_p_^2^ = 0.0832) (Table 6) among African Americans. There were no significant associations between (1) IL SNPs and interleukin trajectory group and (2) IL levels and categorical infant outcomes (data not shown).

## Discussion

This study suggests that maternal SNPs may influence IL milk concentration, and resulting IL milk levels affect continuous NICU outcomes. Our findings are consistent with previous work that demonstrates a functional effect of SNPs on IL concentration for rs1800795,^32^ rs1800796,^18^ and rs1800896.^25,26^ However, this is the first study to reveal this relationship in breast milk. Higher IL6 milk levels were associated with the IL6 -572C allele, and this is consistent with previous studies among acute coronary syndrome patients^18^ and postoperative coronary artery bypass patients.^33^ Our findings are shared with previous findings that show lower plasma IL6 levels among those with the IL6 -174G allele^34^; however, other studies have revealed opposite findings, with higher IL6 levels associated with the G allele among healthy donors,^35^ people with Eales' disease,^36^ and patients in septic shock.^32^ There are also shared findings between IL10 -1082G and subsequent lower IL10 levels, as demonstrated by Qaddourah et al^23^ in serum, and in vitro by liver transplant recipients.^37^ In another study, Capasso et al^26^ demonstrated a different association, with preterm infants who had the -1082G allele experiencing lower IL10 expression. The direction of the SNP's relationship on milk IL levels adds to the already conflicting body of literature. An explanation for these differing findings may be that epigenetic mechanisms also influence IL levels. It is unfortunate that neither this study nor others that examined these SNPs with relation to IL levels included epigenetic mechanisms in their study designs.

Trajectory analyses revealed that infants who received milk from the low-linear IL6 group 1 were more likely to develop IVH than infants who received milk from IL6 group 2. IL6 has been proposed as a strong candidate to modify the risk of perinatal brain injury.^38^ Preterm infants face deficient cerebral structural support and are vulnerable to brain injury. IL6 crosses the blood–brain barrier^39^ and has procoagulative properties.^40,41^ In addition, IL6 activation decreases vitamin K-dependent coagulation factors and subsequent IVH development.^40^ Neurodevelopmental complications among infants who experience IVH are lessened if they received breast milk.^42^ When adequate amounts of IL6 are present in breast milk, infants may receive exogenous coagulative protection against IVH, and neuroprotection to infants who had IVH may last into childhood. IL6 has been observed in high amounts in umbilical vein blood among infants who develop IVH,^43^ although other studies have found no such relationship.^44,45^ Conflicting infant IL6 levels and how they relate to IVH risk, coupled with our results, indicate that exogenous milk IL6 should be examined closely as potentially mediating IVH development.

Although this study was not powered to detect a relationship between IL milk levels and NEC, we were able to detect trending relationships between ILs and infant fecal calprotectin levels. The risk of NEC among preterm infants peaks between 13 and 21 days,^46,47^ and our findings suggest that this risk window is reflected in calprotectin levels. Calprotectin is derived mostly from neutrophils and monocytes,^48^ when white blood cell migration to the intestines activates neutrophils to release this protein.^49^ Fecal calprotectin has been directly associated with inflammation severity in the small intestine, including NEC.^49-52^ Fecal calprotectin is also involved in microbiota establishment in preterm infants,^53^ launching its potential influence on long-term outcomes. The trajectory analysis is consistent with these findings, as lower IL6 milk levels were associated with lower calprotectin levels. This is consistent with the pro-inflammatory role of IL6 in the gut, as illustrated by Hegazy and El-Bedewy,^54^ who ameliorated colitis in vitro by down-regulating IL6. The proinflammatory properties of IL6 include a role in neutrophil transition to monocyte infiltration during inflammation, suggesting a magnified effect when exogenous IL6 (eg, breast milk) is introduced. In vitro IL6 expression is increased in ileum mucosal tissue within the NEC population,^55^ and increased serum IL6 levels reflect the clinical severity of NEC.^56,57^ Higher IL6 levels may predispose the infant to a hyperinflammatory intestinal environment, thereby increasing calprotectin. Cury et al^58^ reported that increased levels of IL6 result in a loss of bowel homeostasis, which may lead to disease development.

Infants who received milk from IL4 group 2 were more likely to receive a blood transfusion when compared with infants who received milk from IL4 group 3. Although all groups are linear in shape, group 3 is higher, suggesting that those infants receive more IL4 via the breast milk. IL4 has a suppressive role on erythropoiesis.^59,60^ This is not consistent with our findings, which suggest that infants who receive less IL4 via breast milk are more likely to require a blood transfusion. This finding should be interpreted cautiously, since the overall effect of trajectory grouping on transfusions was not significant.

Among African Americans, the effect of prepregnancy BMI on milk IL10 levels remained consistent over time, and there were no significant differences in BMI between the 3 ethnic subgroups. Abdominal obesity has been associated with lowgrade inflammation and this increases plasma IL6.^61^ Maternal adiposity has been positively correlated with cord blood IL6 levels in a mixed-race sample,^62^ but a negative correlation was found in a sample of Mexican mothers,^63^ suggesting a race-specific influence of BMI on IL production. In the models for which BMI was significantly associated with milk IL levels, it was an inverse association, suggesting that higher BMIs result in lower IL6 milk concentration.

The effect of maternal BMI on milk IL levels suggests that, in addition to SNPs, environmental factors affect milk composition. Immunofactors present in milk are influenced by weight, including lower milk levels of TGF-β2 and sCD14 levels of overweight mothers when compared with normal weight mothers.^64^ It is estimated that between 50% and 75% of IL10 variability can be exlained by polymorphisms.^65^ Remaining contributors to IL10 production are unknown, although several studies suggest epigenetic influences.^19^ Epigenetic contributors to IL milk levels may explain why we did not uncover more SNP/IL relationships. For example, IL6 milk levels are influenced by mastitis,^66^ preeclampsia,^67^ cesarean section delivery,^68^ and maternal smoking.^69^ In addition, higher IL10 levels are associated with probiotics.^70^ These findings implore an epigenetic approach to understand other molecular mechanisms for breast milk variability.

### Limitations

There were several limitations to this study, most notably a small sample size. Despite trending relationships, the effect sizes ranged from small to medium, and this warrants future studies with larger sample sizes. To control for population substructure and different allele frequencies between ethnicities, we conducted subgroup analyses, which decreased power. The calprotectin analysis should be interpreted cautiously, since calprotectin levels follow their own trajectory patterns.^50^ This is relevant, as we examined cross-sectional calprotectin levels and not patterns over time. In addition, given the small sample size and exploratory nature of this pilot study, we did not correct for multiple comparisons, which may increase Type I error.

Much of this study is based on self-reported variables, including BMI, and study participants underreport weight.^71^ In addition, self-reported ethnicity does not adequately capture inherent biological differences, and ancestral markers are a more reliable way of obtaining biologically relevant information that accounts for admixture.^72^

Hardy-Weinburg Equilibrium was violated for 3 SNPs (rs2070874, rs2243250, rs1800796), although we report significant findings for only 1 of these (rs1800796). We were able to eliminate genotyping error; therefore, we believe HWE violation was due to a biased sample of women who delivered preterm infants, which enriched for the alleles under investigation. The SNPs included in this study have been implicated in a variety of obstetric complications.^73-75^

Infants in this study received donor breast milk, which was not included in the analysis. Of the 73 infants in the study, 31 (42.5%) received donor milk during the first 3 weeks of life (comprising, on average, 10.4%, 11.9%, and 8% of total milk received during weeks 1, 2, and 3, respectively). Infants who receive donor milk are exposed to variable amounts of protein and bioactive components.^76^ Groer et al^77^ measured ILs in breast milk using MAGPIX and found that donor milk retains some IL4, IL6, and IL10. These IL milk levels were much lower in donor milk compared with MOM, likely due to pasteurization and freeze-thaw cycles. This is relevant, as infants in our study were likely exposed to all three ILs from both MOM and donor milk. This could explain our finding that infants who received milk from IL4 trajectory group 2 were more likely to receive blood transfusions than infants who received milk from IL4 trajectory group 3. It is possible that the infants in group 2 received more donor breast milk, and therefore extra IL4, suppressing erythropoiesis; however, we did not measure donor IL concentration. Last, infant serum IL levels were not collected, and this may have better illustrated the potential relationship between exogenous milk IL and infant outcomes.

Future studies should include larger sample sizes to accommodate for additional confounders, including serum infant IL levels. A racially diverse sample size that is larger than the current study would allow for a more robust examination of the effect of minor alleles on subsequent milk IL levels.

## Conclusion

Maternal SNPs may contribute to the immunological profile of breast milk, specifically ILs, which may also affect neonatal outcomes. African American IL milk levels may be influenced by prepregnancy adiposity. In addition, trajectory analyses revealed that IL milk concentration, although linear in shape, is variable in concentration. Future work should examine mechanisms for environmental influences on breast milk.

## Figures and Tables

**Table 1:** Maternal Demographics.^a^

Characteristic	No. (%)
Ethnicity	
Caucasian	21 (33.33)
African American	25 (39.68)
Hispanic	13 (20.63)
Asian	2 (3.17)
Other	1 (1.59)
Education	
Grammar/elementary school	4 (6.25)
Middle school	6 (9.38)
High school	36 (56.25)
College graduate	14 (21.88)
Postgraduate degree	4 (6.25)
Delivery method	
Vaginal	15 (23.44)
Cesarean section	49 (76.56)

a N = 64.

**Table 2:** Infant Demographics.^a^

Characteristic or Outcome	No. (%)
Gender	
Male	38 (52.05)
Female	35 (47.95)
ROP	13 (19.12)
BPD	4 (5.56)
Sepsis	10 (14.08)
NEC	3 (4.17)
IVH	9 (12.86)
Had a blood transfusion	33 (45.21)
Had feeding intolerance	15 (21.13)

Abbreviations: BPD, bronchopulmonary dysplasia; IVH, intraventricular hemorrhage; NEC, necrotizing enterocolitis; ROP, retinopathy of prematurity.

a N = 73.

**Table 3:** Regression Model for Significant Interleukin Concentration with Genotype/Minor Allele Presence Adjusted for Gestational Age at Delivery and Prepregnancy BMI.^a^

SNP/Subgroup	Outcome	Predictor	Estimate	*P* Value	Semipartial ω_p_^2^
1800795					
Caucasian	1nIL6 week 3	MAP–no	−1.134	.097^b^	0.1002
		MAP–yes (reference)			
		Prepregnancy BMI	−0.013	.785	−0.0437
African American	1nIL6 week 1	MAP–no	0.510	.450	−0.0272
		MAP–yes (reference)			
		Prepregnancy BMI	−0.086	.041^c^	−0.0002
African American	1nIL6 week 2	MAP–no	0.661	.275	−0.0005
		MAP–yes (reference)			
		Prepregnancy BMI	−0.069	.064^b^	−0.0401
African American	1nIL6 average	MAP–no	0.498	.405	−0.0186
		MAP–yes (reference)			
		Prepregnancy BMI	−0.096	.013^c^	0.0453
1800796					
African American	1nIL6 week 2	MAP–no	−1.571	.077^b^	0.0477
		MAP–yes (reference)			
		Prepregnancy BMI	−0.076	.029	−0.0040
1800871					
African American	1nIL10 week 1	MAP–no	−0.57	.388	−0.0046
		MAP–yes (reference)			
		Prepregnancy BMI	−0.082	.060^b^	0.0677
African American	1nIL10 week 2	MAP–no	−0.221	.712	−0.0343
		MAP–yes (reference)			
		Prepregnancy BMI	−0.091	.035^c^	0.0986
African American	1nIL10 average	MAP–no	−0.231	.675	−0.0252
		MAP–yes (reference)			
		Prepregnancy BMI	−0.09	.026^c^	0.1325
1800872					
African American	1nIL10 week 1	MAP–no	−0.883	.242	0.0064
		MAP–yes (reference)			
		Prepregnancy BMI	−0.099	.026^c^	0.0285
African American	1nIL10 week 2	MAP–no	−0.769	.247	0.0167
		MAP–yes (reference)			
		Prepregnancy BMI	−0.099	.025^c^	0.0978
African American	1nIL10 average	MAP–no	−0.68	.259	0.0134
		MAP–yes (reference)			
		Prepregnancy BMI	−0.099	.015^c^	0.1189
1800896					
African American	1nIL10 week 3	MAP–no	1.325	.071^b^	0.1145
		MAP–yes (reference)			
		Prepregnancy BMI	−0.082	.175	0.0424

Abbreviations: BMI, body mass index; MAP, minor allele presence.

a N = 64.

b *P* ≤ .10.

c *P* ≤ .05.

**Table 4:** Regression Model for Categorical Infant Outcomes with Interleukin Trajectory Group Adjusted for Gestational Age and Ratio of Mother's Own Milk to Total Milk Administered (Total Population).

Outcome	Predictor	Odds Ratio (95% Confidence Interval)	*P* Value
Sepsis	IL4 group membership		.273
	IL4 group 1 versus 3	0.132 (0.004-4.028)	.142
	IL4 group 2 versus 3	1.050 (0.086-12.818)	.259
Blood transfusion	IL4 group membership		.192
	IL4 group 1 versus 3	1.712 (0.298-9.837)	.796
	IL4 group 2 versus 3	4.162 (0.778-22.277)	.071^a^
Intraventricular hemorrhage	IL6 group membership		.041^b^
	IL6 group 1 versus 2	6.275 (1.076-36.584)	.041^b^

a *P* ≤ .10.

b *P* ≤ .05.

**Table 5:** Regression Model for Continuous Infant Outcomes with Interleukin Trajectory Group Adjusted for Gestational Age and Ratio of Mother's Own Milk to Total Milk Administered (Total Population).

Outcome	Predictor	Estimate	*P* Value	Semipartial ω_p_^2^
lnCalprotectin week 3	Interleukin 6 trajectory group 1	−0.312	.082^a^	0.0376
	Interleukin 6 trajectory group 2 (reference)			
	Interleukin 10 trajectory group			0.0410
	Interleukin 10 trajectory group 1	−0.391	.116	
	Interleukin 10 trajectory group 2	−0.006	.978	
	Interleukin 10 trajectory group 3 (reference)			

a *P* ≤ .10.

**Table 6:** Regression Model for Continuous Infant Outcomes with Interleukin Concentration Adjusted for Gestational Age at Delivery and Ratio of Mother's Own Milk to Total Milk Administered.

Subgroup	Outcome	Predictor	Estimate	*P* Value	Semipartial ω_p_^2^
Caucasian	LnCalprotectin week 3	lnIL6 week 3	0.118	.328	0.0017
	Weight at 6 weeks	lnIL6 week 3	−0.01	.707	−0.0039
	Length of stay	lnIL6 week 3	−2.014	.521	−0.0197
	Days on oxygen	lnIL6 week 3	2.894	.328	0.0004
	lnSNAP final	lnIL6 week 3	−0.027	.813	−0.0527
African American	LnCalprotectin week 2	lnIL6 week 2	−0.015	.892	−0.0401
	LnCalprotectin week 3	lnIL6 week 2	0.137	.098^a^	0.0832
	Weight at 6 weeks	lnIL6 week 2	−0.006	.801	−0.0266
	Length of stay	lnIL6 week 2	3.717	.285	0.0032
	Days on oxygen	lnIL6 week 2	−1.051	.706	−0.0240
	lnSNAP final	lnIL6 week 2	0.017	.770	−0.0329
	lnCalprotectin week 3	lnIL10 week 3	−0.066	.578	−0.0341
	Weight at 6 weeks	lnIL10 week 3	−0.039	.156	0.0306
	Length of stay	lnIL10 week 3	−3.373	.316	0.0009
	Days on oxygen	lnIL10 week 3	4.259	.126	0.0410
	lnSNAP final	lnIL10 week 3	0.002	.965	−0.0339

a *P* ≤ .10.
